# Practical Recommendations for Youth Care Professionals to Improve Evaluation and Reflection During Multidisciplinary Team Discussions: An Action Research Project

**DOI:** 10.5334/ijic.5639

**Published:** 2022-03-30

**Authors:** L. A. Nooteboom, E. A. Mulder, R. R. J. M. Vermeiren, J. Eilander, S. I. van den Driesschen, C. H. Z. Kuiper

**Affiliations:** 1 LUMC Curium – Child and Adolescent Psychiatry, Leiden University Medical Center. Post Box 15, 2300 AA Leiden, The Netherlands; 2 Academic Workplace Youth At Risk, Intermetzo-Pluryn, Post Box 53, 6500 AB Nijmegen, The Netherlands; 3 Department of Child and Adolescent Psychiatry, Amsterdam University Medical Centre – Location VUMC, De Boelaan 1117, 1081 HV Amsterdam, The Netherlands; 4 Youz: Parnassia Group, Dr. van Welylaan 2, 2566 ER The Hague, The Netherlands; 5 Leiden University of Applied Sciences, Zernikedreef 11, 2311 CK Leiden, The Netherlands; 6 iHUB, youth care, special education and youth mental health care Mozartlaan 150, 3055 KM Rotterdam, The Netherlands

**Keywords:** evaluation, reflection, action research, interprofessional collaboration, multidisciplinary, integrated care

## Abstract

**Introduction:**

Integrated care for children and their families is often organized in multidisciplinary teams. In these teams, evaluation and reflection during Multidisciplinary Team Discussions (MTDs) are fundamental to learning, improving interprofessional collaboration, and increasing the quality of care. The effectiveness of MTDs varies widely in practice. Therefore, this study’s objective was to identify facilitators and barriers for evaluation and reflection in MTDs, and concurrently formulate practical recommendations for professionals to improve their MTDs.

**Methods:**

This study’s action research cycle consisted of a qualitative component to identify facilitators and barriers to evaluation and reflection in MTDs. We observed MTDs in multidisciplinary teams and interviewed professionals, parents, managers, and local policy makers. Concurrently, practical recommendations were iteratively developed during project team meetings, learning sessions, and a focus group.

**Results:**

Nine practical recommendations were formulated based on the identified facilitators and barriers, including preparatory activities to ensure purpose, timing, and relevant stakeholder involvement; specific points of attention during MTDs to ensure effectiveness; and tracking follow up steps after MTDs to ensure a learning process.

**Conclusion:**

The practical recommendations should be incorporated in daily practice to support professionals in Youth Care to increase satisfaction and improve effectiveness of evaluation and reflection during MTDs.

## Introduction

Children and their families who receive support from Youth Care services all too often experience a combination of psychosocial-, emotional-, cognitive-, or stress-related impairments, impacting several life domains (e.g., at home, school, and in the community). The needs of these families exceed the expertise and possibilities of a single professional discipline or organization [1]. Hence, multiple professionals from a wide range of Youth Care services are involved in a family’s care process, from universal and preventive services like social work and parenting support, to specialized services such as specialized mental health care and child protection services [2]. To overcome fragmentation in support for these families, organizing integrated care is a necessity [3,4]. Integrated care can be defined as coordinated, coherent and continuous support, aligned across life domains, and tailored to the needs of families [4]. Previous research has shown that integrated care can lead to improved clinical outcomes, increased (cost)-effectiveness of care, and enhanced client satisfaction [4,5].

An important aspect of integrated care is interprofessional collaboration: a process of professionals with complementary backgrounds, who work together to achieve common goals and solving complex issues [6,7,8]. The intensity of interprofessional collaboration varies per case, from sharing brief information and consultation, to collaboratively identifying problems and developing shared care plans [9]. To facilitate interprofessional collaboration, integrated care is often organized in multidisciplinary teams. Multidisciplinary teams refer to a group of professionals that bring together a variety of expertise and skills to jointly assess, plan, and manage care [8]. The composition of a multidisciplinary team depends on families’ needs, and can include professionals representing community work, social work and education, specialized mental health care, parenting support, financial support, and child protection.

Yet, a major challenge to provide integrated care in multidisciplinary teams is that these professionals frequently hold different views, adopt diverse working approaches, or lack collaboration [6,10]. As a result, it can be difficult to jointly prioritize and decide on the focus of support for families. Moreover, since the needs of families often differ across life domains and change over time, professionals must be flexible in their approaches, roles, and responsibilities [10,11]. In order to tailor support to families’ changing needs, mutual coordination among the family and professionals involved is needed. In multidisciplinary teams, this mutual coordination can be achieved by frequent evaluation and reflection on the care process during Multidisciplinary Team Discussions (MTDs) [4,12,13,14].

## Background

Evaluation is conceptualized as systematically monitoring, collecting, discussing, and interpreting information with the intention to appraise the value and effectiveness of a process, plan, or outcome [15]. Reflection on the other hand, is a structured approach to gain insight in one’s own thoughts, values, and behaviors, based on previous experiences. It is a process of looking back on action, increase awareness on essential aspects, feelings and behavior, to find a deeper meaning and improve professional competency [16]. Evaluating care processes and reflecting on prior experiences from a multidisciplinary view are both fundamental to learning and professional development, and can lead to enhanced quality of care and improved working approaches of professionals [10,16,17].

In multidisciplinary teams, evaluation and reflection generally take place during Multidisciplinary Team Discussions (MTDs; [13,14]). MTDs are regularly (often weekly) held team discussions and defined as a moment of both individual and collaborative learning. During MTDs professionals evaluate and reflect in a group on for example: (1) the care process of families, (2) interprofessional collaboration within and outside their multidisciplinary team, or (3) one’s own working approach [13]. Evaluation and reflection during MTDs can improve shared decision making and increase insight in a care process, leading to better outcomes for people in care [8,18]. Moreover, evaluation and reflection in MTDs can lead to improved interprofessional collaboration, by taking advantage of the broad expertise of a multidisciplinary team, developing a common vision and language between professionals, redefining roles and responsibilities if needed, and reducing fragmentation of care [19].

Although there are several working methods available for evaluation and reflection in MTDs [20], the implementation, effectiveness, and efficiency of these working methods varies widely across settings and teams [14,17]. In that, a major barrier is the broad diversity of professional disciplines involved in MTDs. Albeit needed to provide integrated care, this diversity can also lead to misunderstanding of each other’s working approach, a lack of purpose, and less effective decision making during MTDs [13,14,18]. Also, evaluating and reflecting on a broad range of topics in a limited amount of time can lead to a lack of purpose and structure, a lack of in-depth discussion, and inconsistent documentation of decisions during MTDs [14]. Particularly in Youth Care, these barriers might hinder the effectiveness of evaluation and reflection. After all, in Youth Care there are various professional disciplines involved in MTDs, and professionals often discuss a broad range of problems that families in Youth Care encounter [13]. Hence, to achieve effective evaluation and reflection in MTDs, it is necessary to meet certain preconditions [17].

Previous research in adult mental health care led to 21 recommendations to improve the effectiveness of MTDs [17]. These recommendations include the importance of a goal-oriented working approach, clear documentation of outcomes of the MTDs, and sufficient chairing of the session. Nevertheless, these recommendations were constrained to evaluations of single adult interventions and their treatment plan implementation, whereas in integrated Youth Care, professionals support multiple family members with a variety of problems across life domains. To our knowledge, there is a lack of practical recommendations to guide Youth Care professionals in multidisciplinary teams in improving evaluation and reflection during their MTDs. Therefore, this study’s objective was to identify facilitators and barriers for evaluation and reflection in MTDs, and concurrently to formulate practical recommendations in collaboration with professionals from multidisciplinary teams, their managers, local policy makers, and families in Youth Care.

## Method

### Setting

In 2015, the Youth Care system in the Netherlands was decentralized. Ever since, municipalities are responsible for organizing and providing Youth Care on a local level, including preventive health services, youth mental health services, and specialized Youth Care [2]. The aim of this local organization was to improve integrated support at an earlier stage, within the family’s own environment, and with easy access to a variety of services in Youth Care [2]. To achieve this aim, municipalities formed local multidisciplinary teams, called Youth Teams. In these teams, professionals represent various services and expertise, including social work and education, specialized mental health care, infant mental health care, support for youth with (mild) intellectual disability, parenting support, and child protection. Youth Teams operate locally in a primary care setting as a linking pin between preventive services and specialized Youth Care [2]. Professionals in these Youth Teams provide ambulatory support to children (aged 0–23) and their families with a broad variety of psychosocial, stress-related, and socio-economic problems. They focus on strengthening families’ own capacities, involving families’ social network, and coordinate support in collaboration with other (local) services. In this study, six local multidisciplinary teams from the Western part of the Netherlands (Holland Rijnland and The Hague) participated.

### Study design

**Figure 1:**
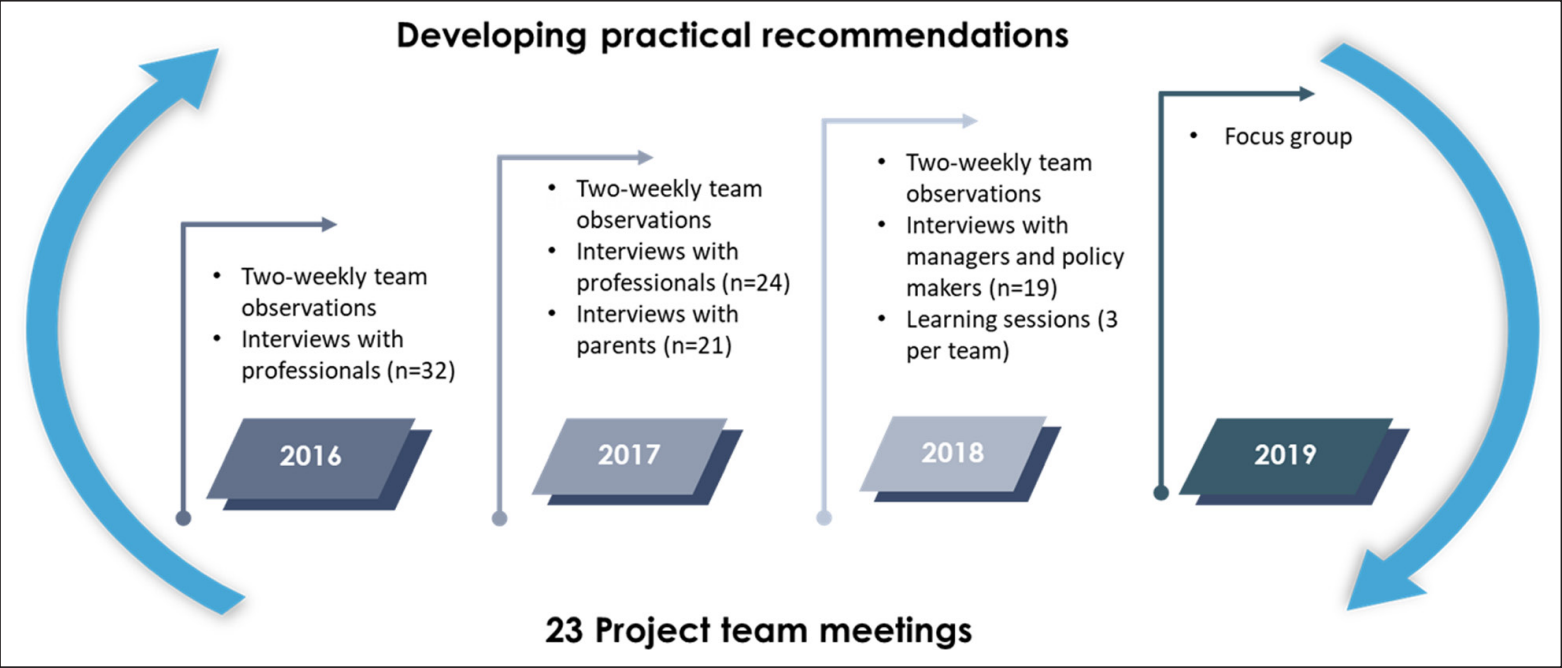
Study design.

This study was part of a four-year research project of the Academic Workplace ‘Gezin aan Zet’ (Family’s Turn). The Medical Ethics Review Board of Leiden University Medical Centre decided that the research project complied with the Netherlands Code of Conduct act for Research Integrity (number P17.018). The study approach was derived from action research, a community-based research method enabling broad understanding of complex processes in practice, while engaging all stakeholders in the research process [21,22]. Hence, action research enhances the validity and applicability of study outcomes [23]. The current study’s action research cycle consisted of a qualitative component to identify facilitators and barriers to MTDs from multiple perspectives (i.e., by interviews and observations; [24]). Concurrently an iterative process of formulating, discussing, implementing, evaluating, and adapting practical recommendations based on the identified facilitators and barriers took place (i.e., by project team meetings, learning sessions, and a focus group [22]). An overview of the study design can be found in ***Figure 1***.

In the following section, the qualitative component is described first, followed by a description of the iterative process of developing practical recommendations. Completeness and reporting quality of the practical recommendations were improved by complying with the Reporting Items for practice Guideline in HealThcare (RIGHT) statement [25].

### Qualitative study

Semi-structured interviews and observations were conducted to identify facilitators and barriers to evaluation and reflection in MTDs.

#### Interviews

Youth Team professionals were the intended primary users of the practical recommendations developed in this study. However, parents, managers, and local policy makers can also participate in MTDs. Hence, to include relevant perspectives on barriers and facilitators, we interviewed the following participants in four different rounds: 4–6 professionals from each of the six participating Youth Teams (in 2016, n = 32; in 2017, n = 24), parents receiving support from Youth Teams (2017, n = 21), and managers and local policy makers involved with the Youth Teams (2017–2018, n = 19). ***Table 1*** provides an overview of the study sample and the various participant characteristics such as age, profession and work experience.

**Table 1:** Demographic characteristics of participants.


VARIABLE	PROFESSIONALS R1 (N = 32)	PROFESSIONALS R2 (N = 24)	PARENTS (N = 21)	MANAGERS AND POLICY MAKERS (N = 19)

Interview duration min [m (range)]	49 (35–60)	56 (39–79)	53 (31–90)	48 (41–60)

Gender [n (%)]				

Male	2 (6.3%)	2 (8.3%)	4 (19.1%)	1 (5.3 %)

Female	30 (93.7%)	22 (91.7%)	17 (80.9%)	18 (94.7%)

Age in years				

Mean age in years (SD)	39.00 (9.13)	39.25 (11.04)	43.75 (8.47)	47.37 (9.38)

Age range in years	24–61	24–61	26–57	28–61

Cultural Background [n (%)]				

Western			17 (85.0%)	

Non-Western			3 (15.0%)	

Highest Educational Level [n (%)]				

Primary Education			2 (10.0%)	

Intermediate Vocational. Educ.			8 (40.0%)	

Higher Vocational. Educ.	24 (75.0%)	21 (87.5%)	7 (35.0%)	9 (47.4%)

University	8 (25.0%)	3 (12.5%)	3 (15.0%)	10 (52.6%)

Study [n (%)]				

Socio-pedagogical assistance	10 (31.2%)	11 (45.8%)		

Pedagogics	8 (25.0%)	6 (25.0%)		

Psychology	3 (9.4%)	1 (4.2%)		

Social work	7 (21.9%)	5 (20.8%)		

Other	4 (12.5%)	1 (4.2%)		

Profession [n (%)]				

Manager				4 (21.1%)

Coach				4 (21.1%)

Policy maker				7 (36.8%)

Staff advisor				2 (10.5%)

Other				2 (10.5 %)

Years of work experience				

Mean years of experience (SD)	15.98 (8.78)	14.23 (9.67)		

Range years of experience	3–39	1.5–35		

Marital Status [n (%)]				

Two-parent household			10 (50.0%)	

Divorced			9 (45.0%)	

Single-parent household			1 (5.0%)	

Number of children [n (%)]				

One child			5 (25.0%)	

Two or more children			15 (75.0%)	

Missing (n)			1	


The professionals, managers, and local policy makers were recruited during MTDs or individually by email by one of the researchers. Convenience sampling was applied based on availability and there were no further in- or exclusion criteria. Parents were invited to participate in a semi-structured interview by an email from their Youth Team professional. To prevent convenience sampling bias, professionals were encouraged to approach all parents in their caseload. Moreover, professionals were asked to search for a diverse sample (i.e., various cultural backgrounds, and both positive and negative experiences with the support provided). To ensure parental perspectives were based on actual experiences, we purposively included parents with at least three visits to a Youth Team professional. Participation was voluntary, and all participants were informed on the aim and procedure of the interviews by means of written informed consent. The interviews were conducted by one of the researchers (LN or JE) together with a student of the Leiden University Medical Center.

The interviews were guided by topic lists, adjusted to the group of participants. The topics were based on previous studies to MTDs [17,18]. The topic lists for professionals slightly differed between the two rounds: in 2016, the focus was on facilitators and barriers of working in multidisciplinary teams, while the 2017, the topic list specifically focused on facilitators and barriers of evaluation and reflection during MTDs. Ten professionals who were interviewed during the interview first round, also participated in the second round. We controlled for this duplication during the analysis by combining the insights from each interview round per individual participant.

The topic list for parents was formulated in collaboration with a parent representative. It included questions regarding the collaboration between professionals and parents, parental involvement in shared decision making, evaluation of the care process, and interprofessional collaboration. The interviews with managers and local policy makers captured general aspects of evaluation, reflection, interprofessional collaboration, and integrated care. To avoid interpretation bias, all interviews were audio-recorded and transcribed verbatim afterwards [26]. No participant expressed interest in commenting on the transcripts.

#### Observations of MTDs

Between 2016 and 2018, two researchers (LN and JE) independently conducted non-participant, unstructured observations of existing MTDs in the six participating Youth Teams [27]. The six teams, each consisting of eight to twelve professionals from various organizations, held similar compositions and tasks. The observations took place twice every month, and each observation had a duration of approximately 2 hours. Field notes were taken, including notes on the preparation, structure, and participants of the MTDs, roles and professional behaviour during the MTD, types of cases discussed, and documentation of decision making. After each observation, field notes were discussed (JE and LN) and summarized in an online logbook for further analysis.

#### Analysis

The interview transcripts and observation summaries were imported into Atlas.ti (v7). Atlas.ti is a commonly used computer program for labelling and organizing text content in qualitative research. To identify facilitators and barriers that might influence the effectiveness of evaluation and reflection in MTDs, a thematic content analysis was conducted [28]. Facilitators were conceptualized as components enabling professionals to perform evaluation and reflection in MTDs. Barriers were defined as components limiting professionals to perform evaluation and reflection in MTDs. The analysis included the following steps: familiarization with the data by reading the transcripts, identifying major themes, coding, charting, mapping, and interpretation [29]. Open coding was applied to all transcripts by two of the researchers (LN, and JE or SvdD). To control for potential differences between teams or stakeholders when merging the coded fragments from various sources (charting), we also labelled the source of each fragment. No interrater reliability was calculated since previous research points out that interrater reliability in coding segments seems ineffective for reliability purposes [30]. In general, there was agreement in coding between the researchers apart from some lingual differences.

In order to formulate generic recommendations, we aimed to find consensus between different stakeholders’ perspectives on barriers and facilitators. Therefore, we searched for corresponding elements (barriers and facilitators) in the analysis, by systematically comparing themes across participants and sources (i.e., interviews or observations). Barriers and facilitators were registered as corresponding elements when described by at least three participants (i.e., professionals, parents, local policy makers, or managers), or when occurring in both an interview transcript and observation summary. Differences in coding were discussed during meetings with the authors, in which an independent researcher (EM) aimed to find consensus between the coders. To limit possible adverse effects of prejudices, the data was interpreted back and forth as an iterative process and supplemented by reflective discussions of the researchers (LN, SvdD, and JE; mapping and interpretation).

### Iterative process to develop practical recommendations

Based on the facilitators and barriers identified in the qualitative part of this study, practical recommendations were concurrently formulated, discussed, applied, evaluated, and adapted in project team meetings, learning sessions, and a focus group. These activities not only encouraged discussion to reveal multiple perspectives, but also improved the applicability and implementation of the results in practice [31].

#### Project team meetings and the steering committee

Between 2016 and 2019, 23 project team meetings took place in which study progress and preliminary recommendations were discussed. In the project team meetings, four professionals, a parent representative, two managers, and four researchers (EM, LN, JE, and SvdD) closely collaborated. The meetings were led by an independent and experienced action researcher (CK) and guided by an agenda that was formulated in advance. During the project team meetings, the identified facilitators and barriers were presented by the researchers (LN, SvdD, JE). In various project team meetings, the project team members formulated preliminary recommendations based on these themes. The project team strived to find consensus by an iterative course of action and informal decision making on the content of the preliminary recommendations. After each project team meeting, the field notes taken by one of the researchers (LN, JE, or SvdD) were summarized and verified by all project team members. Actions originating from these meetings (e.g., adapt recommendations, implementation activities, inform practice) were applied and evaluated in the following meeting.

Alongside this project team, an external steering committee advised the researchers twice a year, for example by reviewing the preliminary recommendations. The steering committee consisted of a professor in child psychiatry (RV), six local policy makers (from The Hague and Holland Rijnland), four representatives from University (Leiden University Medical Center, The Hague University of Applied Sciences, Leiden University of Applied Sciences), a representative of TNO (independent Dutch research organization), and a parent representative.

#### Structured learning sessions

In 2018, the six Youth Teams participated in three structured, team-based, learning sessions. The function of these learning sessions was twofold: (1) to reflect on the preliminary findings and thereby stimulate in depth interpretation and a learning process in practice, and (2) a member check to validate the conceptual formulation of the recommendations [32]. One of the researchers (JE or LN) moderated the learning session, the other took notes for the written summary. A week before each learning session, professionals received a factsheet with preliminary recommendations, based on the facilitators and barriers that were found in the qualitative study. During the learning sessions, professionals reflected on the recommendations by discussing the interpretation, relevance and applicability. Concurrently, the professionals formulated action points to pilot-test the recommendations in practice, for example by appointing a chair of the MTD or by restructuring the MTD. This pilot-testing process was monitored by the researchers during the MTD observations that followed each learning session (qualitative component). Observations were discussed and interpreted during the project team meetings. The outcomes of the learning sessions were compared to the preliminary recommendations and served as a verification and refinement of the recommendations.

#### Focus group

In 2019, a focus group with 20 professionals from other Youth Teams in Holland Rijnland and The Hague took place. These professionals were unfamiliar with the study and the process of developing practical recommendations. The focus group served both as a member check and as an implementation activity to improve feasibility of the practical recommendations. The focus group was led by a trained moderator (LN) and supported by an observer (SvdD) who took field notes and wrote a summary afterwards. During the focus group, the preliminary recommendations were shared by means of a predefined script and a fictional case to practice with the application of the recommendations. Apart from some linguistic modifications, no major changes were suggested by professionals during the focus group. The recommendations were judged as recognizable and useful, indicating transferability of the recommendations to other multidisciplinary teams in Youth Care.

## Results

All professionals and managers that were interviewed described evaluation and reflection as important processes in their daily work. Moreover, local policy makers and parents confirmed the importance of evaluation and reflection, as it can improve quality of care provided.

“By evaluating, you remain aware that certain expertise might be missing, and that you have to engage other professionals. You get to know your own qualities, and blind spots. To enable this process, you have to stop and reflect on your work.” – Professional HR1.

In general, professionals discussed progression of individual care processes as main part of their MTDs, followed by a shorter discussion of interprofessional collaboration, team development, and regular issues in professionals’ daily practice. Each team had its own working approach, structure, and culture during the MTDs, which varied during the study for example due to changes in team composition or new working approaches. In most observations, professionals held MTDs within their own multidisciplinary team. However, there were also some MTDs in which local policy makers or parents attended the meeting.

### Barriers and facilitators

To identify facilitators and barriers to evaluation and reflection in MTDs, we systematically compared observational data and interview fragments, and compared outcomes from the different stakeholder perspectives. ***Table 2*** presents a detailed list of facilitators and barriers, that were reported by at least three participants during various interview rounds, or reported in both the observations and interviews.

**Table 2:** Recommendations based on facilitators and barriers to evaluation and reflection in Multidisciplinary Team Discussions (MTDs).


	RECOMMENDATION	FACILITATORS	BARRIERS

1	Decide on the subject and goal of the MTD	Clear subject of the MTD (e.g., team process, content of care) Define goal and purpose beforehand	Unclear subject, purpose and focus of the MTD Interchangeably evaluate different subjects during MTDs

2	Differentiate between those involved and those attending the MTD	Decide on those involved and who should attend the MTD Inform all those involved afterwards Availability of professionals MTDs in smaller groups	Too many professionals attending the MTD Lack of sharing information afterwards A broad variety of professional disciplines involved without a clear purpose

3	Decide on the moment and duration of the MTD	Schedule MTDs in advance Sufficient time in between MTDs Estimate duration of each component of the MTD	Not prioritizing MTDs due to a high workload Too lengthy MTDs Too many topics to be discussed in limited amount of time Planning too many MTDs in a short amount of time

4	Timely prepare the MTD and gather input from stakeholders beforehand	Timely and sufficient preparation Collect relevant input from stakeholders	Lack of preparation by those involved in the MTD Lack of input from relevant stakeholders

5	Follow the general structure of MTDs and decide on the working approach	Flexible, shared working approach Time to acquire a working approach Clear format of the MTD An a priori formulated agenda Visualized structure of the MTD Reprise of a preparatory assignment	Rigid working approach that does not fit purpose of the MTD Lack of structure or agenda Variety of working approaches Lengthy decision-making processes

6	Allocate tasks to ensure structured MTDs	Clear allocation of tasks: process guard, chair, secretary, time guard Discussion of tasks and roles beforehand	No secretary Too many tasks for the chair No time guard

7	Ensure a safe team climate during MTD	Open and curious attitude, equality, and mutual respect Clear intentions Room for reflection on limitation and doubts Familiarity Positive atmosphere with focus on learning Appreciation of the multidisciplinary character	Changes in team composition Feelings of dissatisfaction (Negative) consequences after the MTD Interprofessional conflicts Unfamiliarity with those involved in the MTDInequalities between those involved Lack of participation in the MTD

8	Ask reflective questions and provide constructive feedback during the MTD	Objective questions with a focus on learning and improvement Sharing representative information Sufficient time for feedback	Directly provide a solution Focus on negative feedback Focus on incidents outside the context

9	Register and monitor follow-up steps at the end of the MTD	Collaboratively formulate follow up steps (SMART) at the end of the MTD Summary with highlights of the MTD Regularly monitor follow up steps	Lack of time at the end of the MTD Undefined follow-up steps Lack of registration of follow up steps


Overall, the facilitators and barriers reported in the various interview rounds corresponded with the facilitators and barriers observed during the MTDs. For example, according to professionals and during the observations, we found that it was difficult to distinguish the subject, purpose, and focus of MTDs. Moreover, most facilitators and barriers described by parents, managers, and policy makers were also reported by professionals. For example, they all described that a lack of structure and preparation of MTDs led to dissatisfaction and a lack of effectiveness. However, there seemed to be some differences in the consequences that inefficient MTDs might have on different groups of participants. Where professionals generally experienced feelings of demotivation or frustration towards their teams’ working approach during MTDs, parents reported feelings of stress and uncertainty towards their care process after inefficient MTDs.

Another finding from both the interviews and the observations was that too many professionals attending the MTD decreased the effectiveness of the MTD. Especially in case there was a broad variety of professional disciplines involved, this led to prolonged MTDs with too many topics to be discussed in a limited amount of time, an unsafe team climate, parental stress, and lengthy decision-making processes. Interestingly, policy makers were often unaware of the impact of their attendance in MTDs on feelings of unsafety by experienced by professionals. For example, some professionals felt controlled and the urge to defend themselves during these MTDs, while policy makers usually came to learn from professionals’ daily practice. Hence, it deemed crucial that policy makers explicitly discussed their intention of attending an MTD in advance, to increase feelings of safety during MTDs.

### Practical recommendations

The iterative process of formulating recommendations based on the facilitators and barriers led to nine practical recommendations to guide professionals in improving evaluation and reflection during MTDs. These recommendations are listed in ***Table 2***. In the following section, the nine recommendations are described in detail.

#### 1. Decide on the subject and goal of the MTD

Being aware of the goal and subject prior to the MTD can lead to increased feelings of motivation, effort, and focus during MTDs of all those involved. In that, professionals should be aware of goals focusing on team processes (e.g., improving interprofessional collaboration, reflect on team functioning) and goals concerning the content of care (e.g., enhance insight in care processes, reflect on client satisfaction, increase awareness of one’s own working approach). Parents and youth should always be informed about the goal and subject of the MTD beforehand.

#### 2. Differentiate between those involved and those attending the MTD

In general, MTDs were reported as more efficient in relative smaller groups. It is not always a necessity that those involved also physically attend the MTD, as long as a summary of the MTD is reported to all those involved afterwards. For example, not all team members should be attending when their working approach is evaluated with policy makers. Moreover, for parents, it can be very stressful when a group of over 15 professionals evaluate their care process. Therefore, they should get the opportunity to decide on who should be involved in their MTD.

#### 3. Decide on the moment and duration of the MTD

MTDs should be scheduled in advance to ensure evaluation and reflection are regularly performed, even during busy periods.

“Clients always come first, which means that good evaluation can be neglected. However, professionals should also pay attention to sharpening their saw.” – Professional HR2.

To stimulate a learning process, implement change, and ensure improvement in practice, professionals should ensure sufficient time in between MTDs. The duration of the MTD should be estimated beforehand and can vary depending on the goal, subject, and size of the group.

#### 4. Timely prepare the MTD and gather input from stakeholders beforehand

Timely preparation of MTDs is crucial to increase the efficiency, effectiveness, and feelings of satisfaction amongst those involved in the MTD. Specifically, MTDs should be prepared by providing sufficient information to those involved in advance. Professionals can apply various methods to collect input for an MTD, for example by means of a questionnaire, in dialogue, or by group discussions. In case of MTDs with parents and local policy makers involved, specific attention should be paid to gathering their input beforehand. Various participants stated that this the responsibility of the Youth Team professionals or their managers.

#### 5. Follow the general structure of MTDs and decide on the working approach

MTDs should always be guided by an agenda. In general, this agenda should include the following general structure of MTDs: (1) introduction of the goals and structure of the MTD, (2) short reprise of the preparatory assignment, (3) in depth evaluation and reflection on a topic, (4) concrete agreements or follow-up steps, and (5) a summary with the highlights of the MTD. The structure of MTDs can be improved by choosing a working approach beforehand. This working approach should be based on a clear and short format that fits the purpose, group, and subject of the MTD (e.g., a SWOT analysis or the Signs of Safety model).

#### 6. Allocate tasks to ensure structured MTDs

Clear allocation of tasks is needed to safeguard the structure of the MTDs and share responsibility among those involved. The four general tasks during a MTD are: (1) a process guard, responsible for planning the MTDs, inform those involved/attending, and send out the preparatory assignments, (2) a chair, guiding the team through the agenda and structure of the MTD, (3) a secretary, writing down the actions and highlights of the MTD, and (4) a time guard, responsible for time monitoring during MTDs.

“A manager can be the conscience of the group during evaluations.” – Professional DH1.

#### 7. Ensure a safe team climate during the MTD

A safe team climate is essential for all those involved to speak out during the MTD, to learn, and improve practice. A safe climate can be recognized by an open atmosphere, in which professionals feel that there is room for reflection on limitations and doubts. To achieve a safe climate, all those involved should hold a basic attitude of equity, mutual respect, integrity, and trust. This can be improved by transparent communication and showing genuine interest in one another. Moreover, a safe team climate can be improved by explicitly discussing the intention of an MTD in advance and by paying attention to eventual changes in the team composition.

#### 8. Ask reflective questions and provide constructive feedback during the MTD

Professionals should ask reflective questions with the intention to discover the underlying considerations of the other, instead of directly proposing a solution. Reflective questioning and constructive feedback does not imply that one should not be critical, as long as the feedback is objective and focused on increasing awareness on one’s own actions, improvement, and learning. The feedback should be on both positive aspects, as on points that need improvement in order to keep those involved motivated.

“You can only learn from each other if you are daring to provide feedback and are able to receive it.” – Manager DH2.

#### 9. Register and monitor follow-up steps at the end of the MTD

There should be sufficient time at the end of the MTD to repeat key lessons and register concrete follow-up steps. In practice, the importance of this step is often overseen. To ensure a learning process, professionals should keep follow-up steps simple and concrete (specific, measurable, achievable, relevant, and time-bound) and regularly monitor these steps by planning follow up evaluations. Specifically, at the end of the MTD the chair should ask the following question to all those attending: ‘What do we expect from who, at what time, and how to we monitor the progression?’.

## Discussion

Our study’s action research resulted in nine practical recommendations for professionals in Youth Care to improve evaluation and reflection during MTDs. The recommendations are based on facilitators and barriers from MTD observations and interviews with professionals, parents, managers, and local policy makers. They include: (a) preparatory activities to ensure purpose, timing, and relevant stakeholders involved; (b) specific points of attention during MTDs to ensure effectiveness (e.g., a shared working approach, clear tasks and roles, a safe team climate, and reflective questioning); and (c) tracking follow up steps after MTDs to ensure a learning process. By closely collaborating with professionals when developing the recommendations, professionals judged the recommendations as recognizable and applicable to existing MTDs. Moreover, professionals reported that applying these recommendations guided them to improve structure, process, and effectiveness of MTDs. Also, it led to increased feelings of satisfaction among those involved in the MTDs, as reported by the professionals during team observations.

An important finding is that although there was informal consensus on the barriers and facilitators, the impact of these barriers and facilitators seem to differ between the type of participants. Specifically, policy makers underline the importance of a safe team climate, but are often unaware of their own role in creating an unsafe team climate. Moreover, both parents and professionals experience feelings of dissatisfaction when MTDs are inefficient. However, professionals can feel frustrated towards their working approach, while parents can feel stressed about the consequences this inefficiency might have on their care process. It is important that all parties involved in MTDs are aware of these potential different consequences, and discuss these differences in order to create a safe team climate.

Our recommendations with a focus on integrated Youth Care generally corroborate with previous research to MTDs in adult mental healthcare [17]. For example, regarding the importance of a clear structure, a chair, and formulating follow up steps after an MTD. However, we also see some differences. In adult mental health care, the practical recommendations mainly focussed on individual patients and reviewing treatment goals and outcomes [17]. In our study, we found that MTDs in Youth Care are not only used to discuss care processes and treatment plans, but also to evaluate interprofessional collaboration within and outside the multidisciplinary team, and to reflect on one’s own working approach. This is in line with other studies, stating that evaluation and reflection are important tools to improve interprofessional collaboration and the quality of integrated care [12,14]. We also found that the effectiveness and efficiency of MTDs was challenged by the broad range of problems that was discussed during MTDs [14,18]. As previous research points out [13,14], discussing such a broad range of topics in a limited amount of time can lead to a lack of purpose, structure, and depth in the MTD. To increase efficiency of MTDs, it is crucial that professionals in Youth Care decide on the subject and goal beforehand. Moreover, all those attending the MTD should be informed about the subject and goal, in order to sufficiently prepare the MTD.

Besides increasing efficiency, those attending an MTD should also be aware of the importance of familiarity and informal contact [13]. MTDs are the opportunity to invest in this familiarity and thereby improve interprofessional collaboration. At the same time, there is a growing pressure on professionals in Youth Care, due to a lack of time and resources. Hence, professionals should organize MTDs in the most efficient way, while also considering sufficient time for reflection and more informal, interprofessional contact moments. To avoid that MTDs lack focus, we advocate that professionals plan weekly MTDs with a strong focus on the content of care (i.e., families’ care processes) and schedule monthly MTDs with a focus on improving interprofessional collaboration and familiarity.

Another important implication is about the number of professionals that should be involved in the MTD. When providing integrated care for children and families with multiple needs, there are often multiple professionals involved. MTDs can improve collaboration between these professionals, since the various working approaches and differences in perspectives can be evaluated during the meetings, and shared decision making can take place. However, corroborating previous research [14,18], we found that the attendance rate of professionals during MTDs should be limited, since too many professionals attending the MTD hinders the effectiveness. Specifically, this can lead to lengthy decision-making progress and an unsafe team climate, in which those attending the MTD do not feel comfortable to speak out or reflect. Unfortunately, there is no golden standard for the number of professionals attending an MTD, since the number of professionals involved varies on families’ needs and the purpose of the MTD. Based on our observations, we suggest an attendance rate of 5–10 professionals from various disciplines is the maximum to still be efficient, depending on the focus of the MTD and the degree of familiarity. Moreover, parents stated that they should always be involved in deciding who is attending their MTD, since they have an overview of relevant professionals. Furthermore, we found that gathering feedback beforehand from all those involved in the MTD, and provide feedback after the MTD, might help to limit high attendance rates during MTDs. Thus, professionals should be aware of involving parents in decision making on the attendants and sharing relevant information after MTDs with those involved.

### Strengths and limitations

The key strength of our study lays in its participatory approach involving professionals from six different teams in Youth Care with a variety of working experience, professional disciplines, and working approaches. Additionally, we included the perspectives of parents, managers, and policy makers. This participant triangulation, together with triangulation in research methods (e.g., interviews, observations, and focus groups), enabled us to gain a rich and in-depth view of facilitators and barriers to evaluation and reflection in MTDs [33]. The non-participant unstructured observations enabled the researchers to study MTDs without predetermined notion [27]. Furthermore, we ensured feasibility and applicability of the recommendations in practice by collaboratively developing recommendations during project team meetings. The focus group with professionals of other multidisciplinary teams in Youth Care enabled us to confirm the credibility, applicability, and transferability of the recommendations in teams who were unfamiliar with the research project. We therefore believe that further application of recommendations in daily practice of Youth Care professionals is realistic and require a minimum amount of time and no additional financial resources to implement.

However, additional implementation activities are needed to improve transferability of the results, and to implement the recommendations in other multidisciplinary teams. As we know from previous research, multiple factors can play a role in the implementation and there is no comprehensive strategy applicable to all settings [34]. Facilitators to implementation in our study were the project team members and steering committee, who also served as ambassadors within their own organization. They had the formal task to involve their colleagues in applying the recommendations in their work processes. Moreover, the members of the steering committee held key functions within their organizations and could therefore easily spread and implement the results of this study. To implement the results in other settings, we recommend designating local implementation ambassadors with the responsibility to inform and support professionals in applying the practical recommendations.

This study also has its limitations. We systematically compared observational data and interview fragments of a multidisciplinary group of professionals and managers, policy makers, and parents. We concluded that most facilitators and barriers corresponded between sources and research methods. However, all project team members and participants in the interviews were related to Youth Teams in the Netherlands. We studied a typical Western setting, and since cultural norms might vary across countries, we cannot conclude that the recommendations are globally applicable. Moreover, no formal consensus methods were used to formulate the recommendations, such as a Delphi method. In future research, it would be interesting to focus on eventual differences between various stakeholders and settings regarding the impact of these recommendations, to improve applicability and transferability.

Importantly, the effect of applying these recommendations on the quality of care should be evaluated through further investigation. Although triangulation of research methods was applied, the effect of each recommendation in practice is still understudied. Moreover, due to the qualitative focus of our study design, we were unable to calculate the strength of evidence for each recommendation. Based on our observations we suggest that the recommendations might be interrelated, however we did not measure the correlation between recommendations and their effect in practice or in which order the recommendations can be best applied. For example, from our study it remains unclear whether professionals should work on a safe team climate first, before discussing the structure of an MTD. Therefore, to measure their effectiveness in practice, implementation and application of the recommendations should be systematically monitored.

### Conclusion

In conclusion, the nine recommendations formulated and implemented in this study are designed to improve effectiveness of evaluation and reflection in MTDs and thereby increase satisfaction among professionals, improve interprofessional collaboration, and eventually strengthen quality of care. Applying the recommendations in the broad field of Youth Care seem of major importance, since MTDs are crucial to evaluate and reflect on care processes, interprofessional collaboration, and one’s own working approach.
